# The contextual awareness, response and evaluation (CARE) diabetes project: study design for a quantitative survey of diabetes prevalence and non-communicable disease risk in Ga Mashie, Accra, Ghana

**DOI:** 10.1080/16549716.2023.2297513

**Published:** 2024-02-07

**Authors:** Swaib Abubaker Lule, Sandra Boatemaa Kushitor, Carlos S. Grijalva-Eternod, Kafui Adjaye-Gbewonyo, Olutobi Adekunle Sanuade, Mawuli Komla Kushitor, Lydia Okoibhole, Raphael Awuah, Leonard Baatiema, Irene Akwo Kretchy, Daniel Arhinful, Ama de-Graft Aikins, Kwadwo Koram, Edward Fottrell, the CARE Diabetes Team

**Affiliations:** a Institute for Global Health, University College London, London, UK; b Department of Community Health, Ensign Global College, Kpong, Ghana; c Department of Food Science and Centre for Sustainability Studies, Stellenbosch University, Stellenbosch, South Africa; d Department of Population Health, London School of Hygiene and Tropical Medicine, London, UK; e Institute for Lifecourse Development, University of Greenwich, London, UK; f Department of Population Health Sciences, Division of Health System Innovation and Research, Spencer Fox Eccles School of Medicine at the University of Utah, Salt Lake City, UT, USA; g Department of Health Policy, Fred Binka School of Public Health, University of Health and Allied Sciences, Ho, Ghana; h Noguchi Memorial Institute for Medical Research, University of Ghana, Accra, Ghana; i Department of Health Policy, Planning and Management, School of Public Health, University of Ghana, Accra, Ghana; j Center for Tropical Medicine and Global Health Research, Nuffield Department of Medicine, University of Oxford, Oxford, UK; k Department of Pharmacy Practice and Clinical Pharmacy, School of Pharmacy, University of Ghana, Accra, Ghana; l Institute of Advanced Studies, University College London, London, UK

**Keywords:** Type 2 diabetes mellitus, Accra, epidemiology, non-communicable diseases, urban poor communities

## Abstract

Diabetes is estimated to affect between 3.3% and 8.3% of adults in Ghana, and prevalence is expected to rise. The lack of cost-effective diabetes prevention programmes designed specifically for the Ghanaian population warrants urgent attention. The Contextual Awareness, Response and Evaluation (CARE): Diabetes Project in Ghana is a mixed methods study that aims to understand diabetes in the Ga Mashie area of Accra, identify opportunities for community-based intervention and inform future diabetes prevention and control strategies. This paper presents the study design for the quantitative survey within the CARE project. This survey will take place in the densely populated Ga Mashie area of Accra, Ghana. A household survey will be conducted using simple random sampling to select households from 80 enumeration areas identified in the 2021 Ghana Population and Housing Census. Trained enumerators will interview and collect data from permanent residents aged ≥ 25 years. Pregnant women and those who have given birth in the last six months will be excluded. Data analysis will use a combination of descriptive and inferential statistics, and all analyses will account for the cluster sampling design. Analyses will describe the prevalence of diabetes, other morbidities, and associated risk factors and identify the relationship between diabetes and physical, social, and behavioural parameters. This survey will generate evidence on drivers and consequences of diabetes and facilitate efforts to prevent and control diabetes and other NCDs in urban Ghana, with relevance for other low-income communities.

## Introduction

Diabetes is a chronic, non-communicable disease (NCD) characterised by an increase in blood glucose concentrations [1]. Of the three main types of diabetes – type 1, type 2, and gestational diabetes – type 2 accounts for over 90% of all people living with diabetes globally [2]. Those with diabetes are at an increased risk of micro- and macro-vascular complications including, but not limited to, neuropathy, vision loss, and stroke, with implications for quality of life, longevity and individual, household and community economics [3–5].

Globally in 2021, one in ten (537 million) adults between 20–79 years of age were living with diabetes [6], rising from 4·7% in 1980 and 8·5% in 2014 [7,8]. Prevalence is higher in urban than rural areas and in high-income compared to low-income settings [2,9,10]. Global prevalence is expected to rise to 10.2% (578 million) by 2030 and to 12.2% (783 million) by 2045 [2,10] with over two-thirds of this rise occurring in low- and middle-income countries (LMICs) [10–12]. Since there may be no warning signs and early symptoms can be non-specific and difficult to recognise, one in two people living with diabetes are unaware of their condition [2].

In Africa, diabetes prevalence has risen significantly over recent decades and an estimated 55 million people are forecasted to have diabetes in Africa by 2045 [6,8,12]. It is estimated that approximately one in 22 adults (24 million) on the continent were living with diabetes in 2021, resulting in nearly 416,000 deaths [6,10]. Relative to other regions, the African continent has the largest proportion of people with undiagnosed diabetes, with 54% (13 million) of adults living with diabetes but unaware of it [6,10]. Estimates of diabetes prevalence in Ghana range from 4.0% to 8.3% [9,13] and, it is expected that prevalence will rise [14].

The increase in diabetes prevalence is driven in part by urbanisation and changes in diet and lifestyles [2,9,15]. Diabetes is associated with physical inactivity, increasing age, overweight or obesity, poor dietary intake, tobacco use, alcohol consumption, and socioeconomic factors such as education, employment status, wealth, and social class [7,9]. The rise in diabetes prevalence is expected to also increase the number of individuals living with other chronic and acute diseases, with profound effects on their quality of life and functioning, leading to poor mental and physical health, premature mortality, increased demand for health services, and high economic costs [7,15,16]. Despite this high and increasing diabetes prevalence, little research focuses on understanding the burden and context of diabetes among adults in most LMICs, including Ghana [17]. Although evidence is consistent on the growing health and economic burden of diabetes and other NCDs in sub-Saharan Africa (SSA), efforts to prevent and control NCDs remain insufficient [18,19]. In addition to the scarcity of dedicated resources, implementing prevention and control strategies in Africa is impeded by a lack of knowledge of previous evidence-based interventions [19].

Behaviours related to diet, physical activity, regular screening and treatment are known to prevent or delay the consequences of diabetes [4]. However, socio-cultural (e.g. social norms, traditions, and customs) and economic factors, as well as awareness of ones’ diabetic status, are important determinants of these behaviours [20]. Previous studies from Ghana showed that perceptions of risk, unwillingness or inability to change behaviour, lack of support, social stigma, poverty, long distance to healthcare facilities, and poor access to healthy foods are some of the barriers to diabetes prevention and management [21–23]. The lack of cost-effective diabetes prevention programmes explicitly designed for Ghanaian populations warrants urgent attention [24]. For interventions to succeed, understanding the local context and knowledge of previous effective interventions is vital [17]. The ‘Contextual Awareness, Response and Evaluation (CARE): Diabetes in Ghana’ project is a mixed methods study in Ga Mashie, Accra, using an epidemiological survey alongside qualitative methods to generate a contextual understanding of diabetes in an urban poor population. The CARE project builds on earlier work on diabetes in Accra, Ghana, by the Regional Institute for Population Studies (RIPS) Urban Health and Poverty Project [24–27]. In this paper, we describe the CARE survey protocol for the quantitative component of the CARE project. The protocol of the qualitative component is described elsewhere.

## The CARE project aim

The CARE project aims to generate data to understand the burden, narratives, socio-ecological drivers, consequences, and responses to diabetes in Ga Mashie and identify opportunities for community-based interventions for diabetes prevention and control. The current paper describes the survey methods for the quantitative component of the CARE Diabetes project; the qualitative study design and methods are described in detail elsewhere.

## The CARE survey objectives

The primary objective of the CARE Survey is to estimate the prevalence and distribution of diabetes and NCD risk factors among adults in Ga Mashie.

The secondary objectives are to:

1. Estimate the prevalence of multimorbidity in Ga Mashie at the individual and household level, as defined by the co-occurrence at least two of diabetes, hypertension or obesity in one or different household members.
2. Estimate the prevalence of other non-communicable diseases and risk factors and their association with diabetes and multimorbidity.
3. Quantify care-seeking behaviours for diabetes care.
4. Estimate associations between contextual factors and risk of diabetes.

## Methods

### Study design, setting and population

The CARE Survey is a cross-sectional epidemiological study whereby trained local enumerators will collect data from households and individuals in Ga Mashie, Accra, and will use Global Positioning System (GPS) to map the commercial and built environment, including the locations of all food and drink outlets, health facilities and physical, religious, and social spaces.

**Figure 1.**
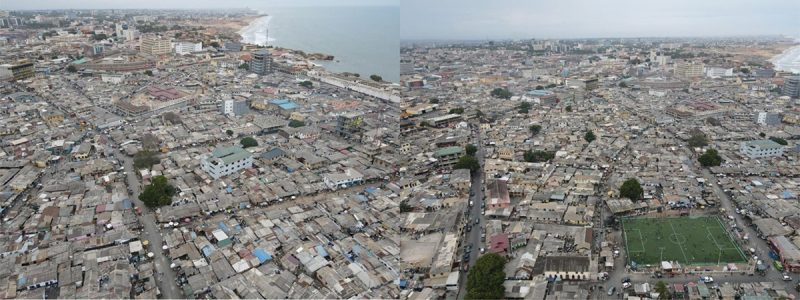
An aerial view of Ga Mashie.

Ga Mashie comprises James Town and Ussher Town across an area of 100 hectares east of the Korle Lagoon on the southwest coast of Accra. It is a densely populated urban setting with a population of around 120,000 and is characterised by low socioeconomic status, low literacy rates, poor sanitary conditions, and predominantly old housing structures [28,29]. Housing consists mostly of double-storey compound houses constructed of sand-concrete blocks and bricks and roofed with aluminium sheeting, slate, or asbestos sheets and arranged into clusters connected via alleyways paved with blocks. On average, over five families reside within a single property; typically, each compound house has 10 to 15 rooms [28,29]. Figure 1 shows the aerial view of Ga Mashie, illustrating the densely populated, urban context.

The area has several formal and informal healthcare providers, including Ussher Town Polyclinic, a government facility [30]. The community of Ga Mashie consists mainly of the indigenous Ga people and migrant populations from other regions [28]. Fishing remains a major source of livelihood, though small-scale trading and other commercial activities now dominate the community [28,31]. London and Salaga markets serve the area, which is important for trade in a wider variety of local food and non-food items. Street trading is commonly engaged in by most households, with food such as Kenkey (a staple dish) often sold in front of many homes [28].

### Study participant inclusion and exclusion criteria

All individuals aged ≥25 years who are permanent residents of the selected households will be eligible to participate in the survey. For the purposes of our study, we define a permanent resident as someone who has lived in a selected household for the past 12 months. A household is defined as a single person living alone or a group of people who may or may not be related but live at the same address and share cooking facilities, a living room, a sitting room, or a dining area [32]. Pregnancy is known to affect blood glucose and blood pressure (BP) and so pregnant women or those who have given birth within the last six months will be excluded from our survey. Additionally, anyone deemed unable to provide informed consent or complete the survey, such as individuals with impaired mental capacity or who are deaf and unaided, will also be excluded.

### Sample size and sampling methods

To calculate the survey sample, we used an assumed diabetes prevalence of 5.0%, a precision of 2.0% and a design effect of 2.5. The assumed prevalence of 5.0% was based on the lowest (thus most conservative) prevalence identified from a review of previous diabetes studies in Ghana [9]. This calculation resulted in a required sample size of 1,242 individuals. To determine the number of households needed for the survey, we estimated that each household would have an average of two eligible adults, with a 10% refusal rate, resulting in a sample of 684 households. However, based on previous field experiences of some of the authors, we further assumed that 40% of households would be empty or non-traceable, leading to an increased sample size of 958 households.

**Figure 2.**
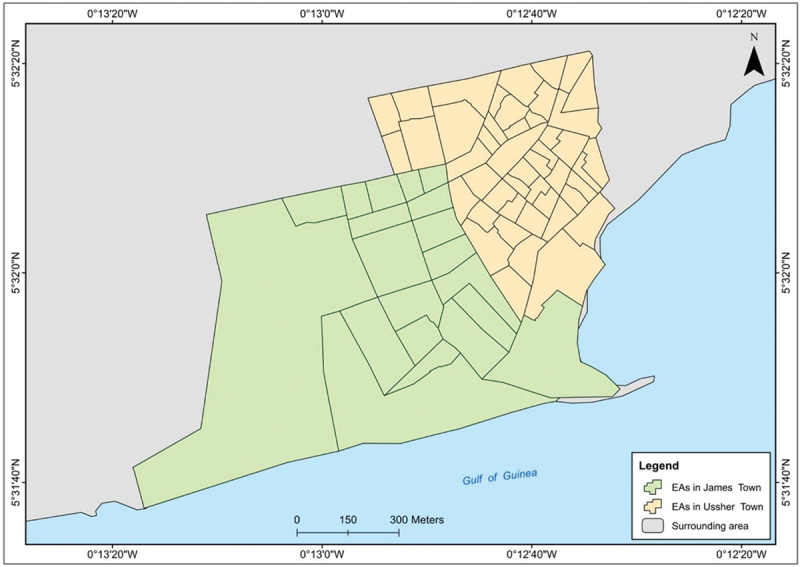
Enumeration area map of Ga Mashie.

We will conduct the survey in the 80 enumeration areas (EAs) of Ga Mashie to ensure a broad geographical representation by including households from all EAs (Figure 2). The Ghana Statistical Service (GSS), which conducted the 2021 Ghana Population and Housing Census (including enumeration of Ga Mashie), will apply simple random sampling to provide a sample of 12 households within each EA for inclusion in this study. As a result, the final sample size will increase to 960 households.

### Preparations

A community engagement activity will be conducted before starting the survey. This will be a participatory event engaging sections of the community, including chiefs, female traditional leaders (often referred to as Queen Mothers), market leaders, fisherfolk, butchers, boxers, and health care providers, among others. The activity will be designed to introduce and explain the project, answer questions, and obtain the views of the potential project participants.

We will partner with the local health authorities to create a health information leaflet about diabetes. The leaflet will describe diabetes, its risk factors, signs, and symptoms, as well as the care and management of the disease. During the survey, we will provide each household with a copy of the leaflet to learn more about identifying, preventing, and treating diabetes and related risk factors. The leaflet will also provide information about where to access related services and treatment.

### Survey team

Forty enumerators will be recruited to conduct the survey. They will undergo a three-day training programme on survey tools and data collection procedures, including obtaining informed consent, conducting participant interviews, maintaining confidentiality, measuring, and recording anthropometric, blood glucose, and BP data, and using the Open Data Kit (ODK) questionnaires on a mobile device. Standard Operating Procedures (SOPs) with detailed procedures to be followed by the survey staff will be developed.

A standardisation exercise will be conducted during the training to ensure reliable anthropometric measurements. The exercise will consist of two sessions, each measuring five adult volunteers in five stations. Each station will have a height board, weight scale, measuring tape, and a mobile device for entering data. During the first session, a group of anthropometric trainees will measure the adult volunteers’ height, weight, and waist circumference twice, at two independent time points. They will move in a clockwise direction to cover all stations. In the second session, the trainees will measure a new set of five volunteers, following the same pattern as in the first session. This approach will help to identify intra- and inter-observer variability and address any apparent inconsistencies or errors in measurement techniques.

### Consent

We will invite all eligible members of the selected households to participate in the survey, and data collection will only start after obtaining informed consent. Enumerators will guide participants through the consenting process by providing information on what the survey is about, the purpose of the survey, what to expect, voluntary participation and discontinuation of participation, benefits and risks for participation, and who is conducting the survey. In this process, enumerators will provide sufficient time to ask questions and provide answers before requesting consent. It is possible that some study participants will be illiterate. In preparation for such instances, enumerators will be trained to verbally explain the study to the participant in the presence of a witness. If the participant understands more than one language, whenever possible, the consent process will be conducted in the preferred language of the participant. In the rare event that the enumerators do not speak any language spoken by the participant, the participant will be excluded and the reason for exclusion recorded. If individuals choose to participate, they will sign the consent form in duplicate and keep one of the copies. A witness will sign in the instance of the participant being illiterate. The remaining copy will be kept securely at the University of Ghana. Participants will receive fresh fruit juice and a cracker, soaps and money as compensation for participating in the survey.

### Procedures

We aim to conduct field activities over six weeks. Individual level data will be gathered from each consenting eligible individual within each household. In addition to providing their own individual-level data, household heads (the main earner or one that is responsible for purchases) as defined by household members will provide data on household level data.

To estimate the prevalence of common NCD risk factors and their association with diabetes and quantify care-seeking behaviours for diabetes, individual and household data will be collected from the respondents. For the purposes of our study, we use the term ‘risk factors’ to refer to personal or contextual factors associated with an increase in the outcomes of interest. Individual-level data will be collected from all eligible participants. We will obtain detailed information on diabetes, other NCDs and related risk factors, awareness, and risk perception. Information on sociodemographic characteristics, psychosocial wellbeing, physical activity, dietary intake, alcohol use, tobacco use, physical activity and exercise, and health service utilisation among study participants will also be obtained. Table 1 summarises the measures taken, operational definitions, rationale and tools to be used.

**Table 1.** List of measures included in the survey, with operational definitions, rationale, level of measurement (household or individual) and tools used.

Measure	Operational definition	Rationale	Household or individual level measure	Tool used
Household characteristics	Household membership, occupation of household head, household asset ownership, household water and sanitation details, household fuel use, size, financial hardship	To characterise the sampled household	Household	Adapted from Ghana Demographic & Health Survey [44]
Individual characteristics	Age, sex, religion, ethnic group, education, occupation, marital status	To characterise the sampled individuals	Individual	Adapted from Ghana Demographic & Health Survey [44]
Social cohesion and trust	Broad concept spanning community sense of belonging, trust in the community, sense of obligation to help others and confidence in return of assistance, equality with community members, and community networks.	To understand aspects of social cohesion and trust and associations with health behaviours and disease outcomes (Objective 4)	Individual	Urban Health and Poverty Survey [45]
Medical history related to the professional diagnosis of the main NCDs (Heart Disease *(Angina, Abnormal Heart Rhythm)*, Stroke, Chronic Lung Disease (*Chronic Bronchitis or Emphysema*), Hypertension, Cancer, Asthma, Arthritis, Kidney Disease, Liver Disease, High Blood Cholesterol, Obesity)	Medical history based on self-report of a diagnosis of condition by a medical professional.	To estimate prevalence of major non-communicable diseases and common risk factors (Objectives 1 & 2).	Individual	WHO STEPwise tool [46] Global Physical Activity Questionnaire (GPAQ) [48]
Risk behaviours including tobacco use alcohol consumption, and physical inactivity.	Physical activity refers to all movement including during leisure time, for transport to get to and from places, or as part of a person’s work.			
Diet quality	Consumption of different food groups, i.e. sets of foods that share similar nutritional properties or biological or culinary characteristics.	To understand dietary practices and quality as a major risk factor for NCDS (Objective 2)	Individual	Diet Quality Questionnaire [47]
Self-rated health	Perceived health status based on a scale of 0 (worst health) to 100 (perfect health).	To understand perceived health status as a risk factor and/or consequence of health behaviours and outcomes.	Individual	Score from 0 to 100
Quality of Life	An individual’s perception of their position in life in the context of the culture and value systems in which they live and in relation to their goals, expectations, standards, and concerns.	To understand associations between quality of life and NCD risk and disease. Quality of life may be a risk for or consequence of diabetes and other NCDs.	Individual	World Health Organisation Quality of Life (WHOQOL-BREF) [41]
Social Support	The number of close confidants, sense of concern from other people, and relationships with neighbours, with a focus on the accessibility of practical help.	To understand the social context and associations with diabetes and other NCD outcomes and risks. (Objective 4)	Individual	Oslo Social Support Scale (OSSS-3) [49]
Personal stress	Extent to which events in a person’s life are assessed as stressful, unpredictable, and uncontrollable.	To understand associations between health, behaviours, and perceived personal stress as a possible risk factor for or consequence of ill health.	Individual	Perceived Stress Scale (PSS-10) [50]
Psychological distress	A measure of stress, anxiety, and depressive symptoms.	To understand associations between health, behaviours, and psychological wellbeing as a possible risk factor for or consequence of ill health.	Individual	Psychological Distress Scale (PDS) developed and used in the Urban Health and Poverty Survey (EDULINK Wave III) survey [45]
Diabetes knowledge, diabetes-related behaviours, and diabetes-related consequences	Knowledge and awareness of the causes, signs/symptoms, consequences, and ways to prevent/control diabetes; self-reported history of diabetes testing and/or diagnosis of diabetes; care-seeking self-reported diabetes-related complications among individuals living with a diagnosis of diabetes	To describe population awareness and knowledge of diabetes and, for those living with the disease, their care-seeking behaviours, and self-reported complications of diabetes.	Individual	Questions developed by the study team and adapted from the Bangladesh DMagic study tools [53]
Appraisal of diabetes among those living with a diagnosis	Perceptions of one’s ability to control their diabetes and its impacts on life goals.	To understand an individual’s thoughts about coping with a diagnosis of diabetes. This will provide insight into the consequences of diabetes in the study context.	Individual	Appraisal of Diabetes Scale [51]
Self-management of diabetes	Perceived self-efficacy of patients to self-manage their diabetes	To understand an individual’s perception of their ability to manage their diabetes through behaviours, medication, etc. within the study context.	Individual	Diabetes Empowerment Scale Short Form (DES-SF) [52]
Care-seeking and costs of care seeking	Self-reported care-seeking and associated costs related to an NCD	To understand care-seeking behaviours and economic consequences of diabetes and other NCDs. (Objective 3)	Individual	Questions developed by the study team.
Weight, height, and waist circumference	Absolute measures to the nearest 0.1 cm for height and waist circumference, and to the nearest 0.1 kg for weight.	To measure adiposity. Obesity will be defined as BMI (weight/height^2^)>30. Central obesity will be defined as a waist circumference ≥102 cm for men or ≥88 cm for women or a waist-for-height ratio > 0.5. (Objectives 1 & 2)	Individual	Measuring tape & weighing scales
Blood pressure	Diastolic and systolic blood pressure in mmHg	To measure blood pressure (Objectives 1 & 2)	Individual	Digital blood pressure monitor
Blood glucose	Random blood glucose in mmol/l	To estimate risk of diabetes where glucose concentration ≥11.1 mmol/L indicates diabetes. (Objectives 1 & 2)	Individual	Digital point-of care glucometer

Blood glucose, BP, and anthropometry (weight, height, and waist circumference) measurements will be used to estimate diabetes, hypertension, and obesity, respectively. We will estimate random blood glucose concentration by using a point-of-care glucometer (One Touch select plus, LifeScan Europe GmbH 6300 Zug, Switzerland) on capillary whole blood obtained from the middle figure following a finger prick. Whilst random blood glucose is not a diagnostic test for diabetes, it is useful for identifying risk of diabetes in population-based surveys where alternative measures such as fasting blood glucose and two-hour glucose tolerance tests are difficult to obtain [33]. As such, we define diabetes in our survey as random glucose concentration ≥11.1 mmol/L [3,34]. Three BP measurements will be taken on the left arm (except where not possible) raised at the heart level with a one-minute interval between measurements after an initial rest, with the participant seated quietly for at least five minutes [35]. An appropriate size cuff on a digital BP monitor (OMRON – M7 intelli IT HEM-7361T-EBK, Vietnam) will be used. The average of the second and third BP measurements will be used to estimate systolic and diastolic BP. Evidence from earlier studies shows that the second and third BP measurements are, on average, more consistent and lower than the first BP measurement [36, 37]. Hypertension will be defined as systolic BP ≥140 mmHg and/or diastolic BP ≥90 mmHg or a self-reported diagnosis of hypertension by a medical professional or self-reported use of antihypertensive medication.

When measuring weight, participants will be asked to stand unsupported on the centre of the scale, with the weight distributed evenly on both feet, arms hanging freely at the sides and wearing light clothing [38]. Weight will be recorded to the nearest 0.1 kg using a digital scale (GLC-D-200 KG digital body scale, GreenLife Canada). Height will be measured with stadiometers to the nearest completed 0.1 cm, with the participant standing with the feet together, without shoes, and the heels, buttocks and upper part of the back projected on the same vertical plane, with the head oriented on the plane of Frankfurt [38]. Waist circumference will be measured at the navel level using a measuring tape to the nearest completed 0.1 cm [38]. The patient will be instructed to breathe normally, and the measurement will be taken at the end of a normal expiration.

BMI will be calculated as weight in kg divided by height squared (m^2^), and obesity will be defined as a BMI ≥30 kg/m^2^ [39]. Central obesity will be defined as a waist circumference ≥102 cm for men or ≥88 cm for women [40] and [42]. A wealth index score will be generated using principal components analysis of items owned [43].

### Data capture, management, and analysis

We will capture household-level and individual-level data using electronic questionnaires preloaded into the Open Data Kit (ODK) Collect app on an Android mobile device. These mobile devices will be encrypted and password-protected.

For each household on the sampling list, a unique household identifier will be generated, barcoded, and uploaded into the ODK. Additional household information obtained from GSS, including the structure number, house number, subjects’ names, telephone number, and address, will also be uploaded in the ODK and used to identify a selected household. The household head will then confirm whether the information about his/her household is correct. If correct, the same unique household identifier will be applied to all members of the same household. Household and individual-level data will be collected separately on different ODK questionnaires for members of the same household. Collected data will be uploaded regularly onto a dedicated secure server for storage, cleaning, coding, and anonymisation.

When a selected household cannot be located or correctly identified (e.g. identification data on the sampling list does not match the information provided by the participants) or the participant refuses or discontinues participation, the enumerators will not proceed with data collection but move to locate and identify the next household on the sampling list.

Specific analysis plans to address the objectives of the CARE Diabetes project will be described elsewhere, and we refrain from defining variables as exposures or outcomes in this study design paper in recognition that 1) a single variable may have various roles in subsequent analyses to understand the complex inter-relationships between contextual factors and health and 2) the cross-sectional design of our survey hinders assessment of cause and effect implied by exposure and outcome labels assigned at the design stage. Broadly, analysis will entail a description of study population characteristics summarised as frequencies and percentages for categorical variables and means with standard deviation or median with 25^th^ and 75^th^ percentile for continuous variables, depending on the nature of the distribution. Chi-square tests will be used for comparison between categorical variables, and we will use parametric or non-parametric tests such as the t-test or Wilcoxon-Mann-Whitney test to test differences between continuous variables, depending on data distribution as appropriate. We will use logistic and linear regression models to further elucidate associations between variables, controlling for potential confounding and effect modification as appropriate and such analyses will be informed by theoretical frameworks and specified in subsequent papers. All analyses will account for the survey design and unequal probability sampling methods applied. Missing data will be treated as missing, without imputation. Data outliers will be identified on the basis of plausibility and probability and any that cannot be corrected will be re-coded as missing values.

### Quality control and validation of the information

Staff for this survey will be trained using SOPs to conduct various procedures, including measurement of BP and anthropometric parameters, consenting, and data collection.

Data will be entered directly in ODK on Android devices. The ODK form will be designed with constraints, logic and consistency checks to ensure data accuracy and completeness. Field team leaders will coordinate and supervise data collection and quality checks. Data cleaning will be conducted to ensure a suitable dataset is available for analysis.

### Pilot testing

Pilot testing of the survey tools and procedures will be conducted among 50 households selected at random by the GSS in the La municipal area of Accra. The same selection criteria will be used for the inclusion and exclusion of participants in the pilot study. Field procedures and data processes will be modified to address any challenges identified in the pilot test and to maximise data quality in the full survey. The time required to complete a home visit will be estimated. Data from the pilot study will not be included in the survey data and will not be considered for data analysis.

### Expected outcomes

The survey will estimate the prevalence of diabetes, hypertension, overweight and obesity, self-reported NCDs and major NCD risks in Ga Mashie. Analyses will identify important associations between socio-demographic, contextual and physical parameters and risk of diabetes and other NCDs. The evidence generated will help build a detailed understanding of the context, socio-cultural and economic drivers, and consequences of diabetes and NCD risk in Accra, including associations with quality of life and healthcare expenditure.

This survey will map the local area to further enhance our understanding of how the physical environment in terms of the commercial, recreational, and social use of space, and perceptions of security can impact diabetes, NCDs and associated risk factors.

Through community engagement initiatives throughout the project and subsequent sharing of study findings with the community, it is anticipated that this project will contribute to community and stakeholder capacity to understand health in this population and organise and advocate for initiatives to prevent and control diabetes and other NCDs.

### Public involvement

The Ga Mashie Development Agency (GAMADA) and the local chief’s council are leading governance organisations within the community of Ga Mashie. GAMADA is an organisation tasked with leading development processes in the two communities that form Old Accra. Approval from these organisations and stakeholders is needed before any research engagement can happen in the community. The CARE project will collaborate with GAMADA, the local chief’s council and the Ghana NCD Alliance, an NGO involved in NCD advocacy. Various stakeholders have been consulted during protocol development and will have opportunities to engage throughout the project.

A community orientation meeting (known locally as a *durbar*) was hosted by GAMADA on 27 August 2022 to formally introduce the CARE diabetes project to the two study communities, James Town and Ussher Town. This was necessary to ensure that the CARE diabetes project activities aligned with the general development goals of the communities. A broad profile of community members attended the community engagement activity, including groups of young people from local boxing clubs and from a local NGO focused on youth development through basketball, and older people from the James Town Health Club (an initiative originating from several years of work by the research team in the community). Many community leaders graced the programme, including chiefs, fishermen, and market leaders. Two representatives from the community’s main public hospital (Ussher Polyclinic) also participated.

## Discussion

We have described the design of a cross-sectional survey to quantify the prevalence of diabetes and other major NCDs and risk factors, care seeking, and the social and contextual factors that are associated with diabetes in a poor urban community in Ghana. This is part of the larger CARE Diabetes project, which seeks to apply transdisciplinary approaches to understand how context influences the burden of diabetes and its consequences in order to develop context-specific intervention strategies. This addresses data gaps on contextual factors driving prevalence, management, and control of diabetes and other NCDs in urban Africa, and our findings will inform the development and evaluation of interventional studies on diabetes and other NCDs in the study setting and elsewhere.

The large, population-based random-sample design using objective measures of blood glucose, blood pressure and anthropometry, and standardised tools for measurement of social, behavioural, and contextual factors are major strengths of our design. Nevertheless, our study design reflects a compromise between the precision of measurements and feasibility of data collection in a population-based survey in a densely populated, poor urban context. For example, we will use random plasma glucose (RPG) to test for diabetes in this population instead of the more sensitive approach of fasting plasma glucose (FPG) on its own or combined with an oral glucose tolerance test (OGTT), which would also allow us to identify instances of impaired fasting glucose and impaired glucose tolerance – stages of intermediate hyperglycaemia associated with increased risk of diabetes. This decision is based on the impracticalities of ensuring fasting in our study sample and the excessive time demands required for OGTT, which demands repeat blood glucose testing two-hours after an oral glucose load. Specifically, FPG in population-based surveys requires multiple contacts with participants who need to be briefed to abstain from all food and drink, except water, for at least eight hours, and requires enumerators to take measures early in the morning before the fast is broken – a time when the majority of our study population will be engaged in commercial activities. Further, the use of FPG and OGTT increases the duration of data collection substantially and would double the number of blood samples that need to be taken, with implications for participants and project resources. Tests would need to be rescheduled if participants failed to abstain from food and drinks, and it is likely that the approach would increase non-compliance, refusal or withdrawal from the survey.

The RPG is a simple, low-cost, and quick procedure that can be done at any time of day and can be completed in a single contact with the participant at their home, with results available immediately. RPG can be influenced by time of test and time since last meal – details which we will record in our survey and will use in interpreting our data. So, whilst RPG is not suitable as a clinical diagnostic test for diabetes, it is a practical tool for epidemiological description of diabetes risk in a population where random plasma glucose levels ≥11.1 mmol/L will be interpreted as screen-positive for diabetes [3,33]. Study participants will be informed that the RPG test will not provide a diagnosis of diabetes, and participants with higher-than-expected RPG levels (≥6.1 mmol/L) will be referred for further assessment at the Ussher Town Polyclinic or Korle-Bu Teaching Hospital. Alternative methods such as HbA1c, dry blood spots or venous blood sampling for laboratory analysis were not considered viable in the study context.

A further limitation of our study design is that several of the parameters measured through our survey rely on self-reported behaviours, medical history, and knowledge. Though we use many standardised and previously validated tools in our survey, there is potential for recall and reporting bias in data gathered in this way. Internal consistency, range and logic checks in our data collection tools will help to identify any obviously spurious responses, but many may be undetectable. Enumerator training, community engagement, as well as our participant information and consent forms, will emphasise confidentiality and the intended use of the data, which may discourage deliberately false responses. Nevertheless, interpretation of all data should be aware of the inherent limitations of the methods used.

## Paper context

The CARE diabetes project is a transdisciplinary study aiming to generate a contextual understanding of diabetes in urban poor communities in Ghana. The CARE diabetes study seeks to provide relevant information to identify opportunities for community-based intervention strategies for diabetes prevention, management, and control in urban poor communities in Ghana. Given the cross-sectional design of the survey, the temporality of events between diabetes and assessed factors cannot be established.
